# Strategies for developing novel therapeutics for sensorineural hearing loss

**DOI:** 10.3389/fphar.2014.00206

**Published:** 2014-09-16

**Authors:** Takayuki Nakagawa

**Affiliations:** Department of Otolaryngology, Head and Neck Surgery, Graduate School of Medicine, Kyoto UniversityKyoto, Japan

**Keywords:** regeneration, cochlea, sensorineural hearing loss, transdifferentiation, dedifferentiation, stem cell

## Abstract

Sensorineural hearing loss (SNHL) is a common disability in the world; however, at present, options for the pharmacological treatment of SNHL are very limited. Previous studies involving human temporal bone analyses have revealed that the degeneration of the cochlea is a common mechanism of SNHL. A major problem for the development of novel pharmacotherapy for SNHL has been the limited regeneration capacity in mammalian cochlear cells. However, recent progress in basic studies has led to several effective strategies for the induction of regeneration in the mammalian cochlea, in accordance with the stage of degeneration. In addition, recent advances in the identification of human deafness genes and their characterization in mouse models have elucidated cellular and/or molecular mechanisms of SNHL, which will contribute to clarify molecular targets of pharmacotherapy for treatment of SNHL.

## BACKGROUND

Hearing impairment has been the most common cause of disability worldwide. Hearing impairment can be subdivided into two types, conductive and sensorineural hearing loss (SNHL). Although conductive hearing loss is largely overcome by surgical treatment, therapeutic options for SNHL are limited to hearing aids and cochlear implants, if the hearing loss has been reversed. In SNHL with acute onset, pharmacotherapy sometimes cures hearing loss. However, pharmacotherapy options for SNHL are very limited. Therefore, researchers have actively sought novel therapeutics for the treatment of SNHL.

Sensorineural hearing loss is largely caused by the degeneration of the cochlea, a sensory organ for hearing. Studies of human temporal bones have revealed that SNHL can be caused by the degeneration of various components of the cochlea (Merchant and Nadol, 2010). Among the various types of cochlear cells, hair cells, which convert sound stimuli to neural signals, have been a central therapeutic target for developing novel therapeutics. The spiral ganglion neurons (primary auditory neurons) transmit auditory stimuli from the cochlea to the central nervous system. Spiral ganglion neurons are crucial for clinical benefits of a cochlear implant, which is a device that improves hearing in deafened patients. A stimulator of the cochlear implant, with an array of up to 24 electrodes, is surgically inserted into the cochlea and directly stimulates spiral ganglion neurons. Therefore, a loss of spiral ganglion neurons undermines the clinical benefits of cochlear implants. For these reasons, spiral ganglion neurons also have been an important therapeutic target for developing pharmacotherapies for SNHL. Beside hair cells and spiral ganglion neurons, there are a number of other cochlear components that are included in a list of possible therapeutic targets. The stria vascularis and spiral ligament in the cochlear lateral wall are among them. These components play key roles in the maintenance of a high concentration of potassium ions in the endolymph, which is necessary for the depolarization of hair cells.

In mammals, the cochlear components described above have virtually no regenerative capacity; this situation is thought to be an obstacle for the development of novel therapeutics for SNHL. However, recent studies have demonstrated that the mammalian cochlea does have the capacity for regeneration, although it is limited. Several challenges for promotion of the limited capacity for regeneration in mammalian cochleae have been overcome. In the mammalian cochlea, researchers have identified stem cell-like cells (Li et al., 2003; Oshima et al., 2007; Taniguchi et al., 2012; Jan et al., 2013). The functional restoration of mammalian cochleae was achieved via several challenging strategies in experimental animals (Izumikawa et al., 2005; Okano et al., 2005; Chen et al., 2012; Mizutari et al., 2013; Tona et al., 2014a). However, the quality of functionality of the regenerated cochleae is not satisfactory. Therefore, new approaches are required for the development of novel therapeutics for SNHL.

Although strategies for the restoration of damaged cochleae are urgently required, more detailed information on therapeutic targets and the development of diagnostic tools are also necessary. Many studies have been focused on morphological findings in human temporal bones, i.e., the following four classical types of SNHL etiology: the loss of hair cells, loss of spiral ganglion neurons, degeneration of the stria vascularis, and combinations (Merchant and Nadol, 2010).

Recent progress in the identification of human deafness genes and their characterization in mouse models have elucidated cellular and molecular mechanisms behind SNHL (Hereditary Hearing Loss Homepage: http://hereditaryhearingloss.org/), which indicate more detailed targets for the treatment of SNHL.

In this review, we focus on strategies for the induction of regeneration in the mammalian cochlea and on new therapeutic targets for SNHL.

## STRATEGIES FOR INDUCTION OF REGENERATION

The sensory hair cell has been the center of attention in studies of the regeneration of inner-ear. Therefore, in this section, the regeneration of hair cells forms the basis of discussion of possible strategies for development of novel therapeutics.

Before hair cells completely disappear from the organ of Corti, the induction of self-repair may be a pragmatic strategy. For this purpose, we propose a two-step process. The first step is the promotion of the survival of hair cells and the second step is the reconstruction of the components of hair cells. Several therapeutic agents that promote survival or protect from cell death have been reported. Some of such candidates have been tested for efficacy and safety in clinical trials (Suckfuell et al., 2007; Nakagawa et al., 2010). Notably, both clinical trials used local application of drugs to the cochlea, which has several advantages including delivery of drugs at high concentrations, but requires surgical invasiveness.

A close relation between hair cell death and c-jun N-terminal kinase (JNK) has been reported in animal experiments (Pirvola et al., 2000; Wang et al., 2003; Murai et al., 2008). Recently, AM-111, a peptide JNK inhibitor, has been examined its potential for prevention of acoustic trauma in humans (Suckfuell et al., 2007). A clinical trial of recombinant human insulin-like growth factor-1 (IGF-1) in sudden deafness has also been performed (Nakagawa et al., 2010), based on the results in animal experiments (Lee et al., 2007; Fujiwara et al., 2008). More recently, mechanisms of IGF-1 actions on the cochlear sensory epithelium have been demonstrated (Hayashi et al., 2013, 2014). In contrast to the promotion of the survival, there are no specific reports describing the induction of the reconstruction of cellular components, such as stereociliary bundles, in damaged hair cells. In this regard, it would be worthwhile to devote more research efforts to the mechanisms underlying the development and maintenance of the stereociliary bundles.

After hair cells are gone, three possible strategies can be utilized depending on the condition of the remaining supporting cells. If sufficient numbers of healthy supporting cells are still present in the organ of Corti, then the induction of the transdifferentiation of supporting cells into hair cells can be a viable strategy. Hair and supporting cells share a common progenitor during development. The manipulation of Notch signaling has been used for induction of transdifferentiation of supporting cells. The transdifferentiation of supporting cells into hair cells was first demonstrated by gene transfer. The introduction of the *Atoh1* gene into supporting cells using an adenoviral vector induces the transdifferentiation of supporting cells into hair cells (Zheng and Gao, 2000; Izumikawa et al., 2005). In theory, the inhibition of Notch signaling upregulates Atoh1. In addition, the pharmacological inhibition of Notch signaling has also been successfully used for this purpose. For example, the inhibition of Notch signaling using γ-secretase inhibitors increases Atoh1 expression in neonatal cochleae, leading to the excessive generation of hair cells (Yamamoto et al., 2006). However, in adult cochleae, no expression of Notch ligands and receptors is virtually detectable in supporting cells. For some time after damage, the temporary activation of Notch signaling is detected even in adult cochleae (Hori et al., 2007; Batts et al., 2009). The topical application of a γ-secretase inhibitor to cochleae results in the induction of hair cell (Hori et al., 2007; Mizutari et al., 2013; Tona et al., 2014a). At present, hearing restoration via this approach is not satisfactory, and the therapeutic time window is narrow. γ-Secretase inhibitors have gained considerable attention because they inhibit Notch signaling and the formation of the β-amyloid peptide. Because of the latter effect, γ-secretase inhibitors have been the main focus of rational drug design in the field of Alzheimer’s disease research. This situation means that various effective γ-secretase inhibitors will soon become available. An extensive search for the best γ-secretase inhibitor for the induction of hair cell is necessary for the clinical application of this strategy.

In the two directions – self-repair and transdifferentiation – pharmacological treatment is expected to become mainstream. For the clinical application of basic research in this field, investigators require drug delivery systems capable of delivering therapeutic molecules to cochlear cells. In parallel with the rational drug design, a lot of research effort in the last decade went into the development of drug delivery systems for the cochlea (Nakagawa and Ito, 2011; Pritz et al., 2013; Staecker and Rodgers, 2013). Among such systems, we clinically applied the gelatin hydrogel, which is suitable for the sustained delivery of peptides or proteins to cochlear cells (Nakagawa et al., 2010). Depending on the chemical characteristics of a therapeutic molecule, the optimal drug delivery system is likely to be different. Therefore, the development of drug delivery systems for specific drugs or biologics should be mentioned among the key challenges for the development of novel treatments of SNHL.

When the number of the remaining supporting cells in the cochlea is insufficient, then the induction of the proliferation in supporting cells is required. In the mammalian cochlea, the proliferation of cell rarely occurs after birth, whereas in the avian cochlea (basilar papilla), supporting cells proliferate in response to hair cell loss (Stone and Cotanche, 2007). In the avian cochlea, both the transdifferentiation of supporting cells into hair cells and proliferation of supporting cells, followed by differentiation into hair cells, takes place (Stone and Cotanche, 2007). To induce the proliferation of supporting cells in the mammalian cochlea, the downregulation of cell cycle inhibitors is a logical step. The major cell cycle inhibitor in mammalian supporting cells is p27, an inhibitor of cyclin-dependent kinase. The genetic deletion of p27 results in the excessive generation of hair cells (Chen and Segil, 1999; Löwenheim et al., 1999). A knockdown of p27 in supporting cells induces re-entry of these cells into the cell cycle, but the majority of the supporting cells that have re-entered the cell cycle then undergo apoptosis (Ono et al., 2009). Therefore, cell cycle re-entry by supporting cells is not sufficient for the regeneration of hair cells, suggesting that some other characteristics of supporting cells also need to be altered. Recently, several challenges for the induction of the dedifferentiation of supporting cells have been described (Burns et al., 2012; Lou et al., 2013). In those studies, transcription factors for the generation of inducible pluripotent stem (iPS) cells were utilized for the induction of the dedifferentiation of inner ear cells. However, the effects of iPS factors in the inner ear have not been explored *in vivo*.

Another option when supporting cells are severely damaged is the introduction of exogenous stem cells into the cochlea. The cell transplantation approach to the regeneration of hair cells has gained considerable attention because stem cells are believed to accumulate in the damaged sites and have the potential for the repair of damaged tissues. On the other hand, the migration of transplanted stem cells into a damaged sensory epithelium of the inner ear is rarely observed (Tateya et al., 2003). In addition, because of the high concentrations of potassium, the conditions in the endolymphatic space of the inner ear are not conducive to the survival of a transplant. Recently, the intrinsic stem cell populations in the inner ear have attracted a lot of interest as an alternative to the introduction of exogenous stem cell-derived cells into cochleae (Li et al., 2003; Oshima et al., 2007; Taniguchi et al., 2012; Jan et al., 2013). However, there is no evidence that internal stem cell populations participate in the maintenance of mammalian cochleae. In addition, the number of stem cell-like cells in the inner ear rapidly decreases because of functional maturation. It is necessary to find a way to enhance the self-renewal potential of endogenous stem cells and induce targeted migration of endogenous stem cells to a damaged tissue. Researchers also require methods for steering the differentiation of endogenous stem cells into the cell lineages of interest.

## THERAPEUTIC TARGETS IN SNHL

Recent advances in the identification of human deafness genes and their physiological characterization in mouse models have elucidated precise cellular mechanisms underlying SNHL (Safieddine et al., 2012). Molecules involved in cochlear ionic homeostasis, mechanoelectrical transduction in the hair bundle, or synaptic transmission by the inner hair cells can be targets for the treatment of SNHL.

The stria vascularis and spiral ligament in the cochlear lateral wall perform crucial functions in the maintenance of cochlear ion homeostasis (Patuzzi, 2011). Ion channels and cell–cell junctions in the stria vascularis and spiral ligament participate in the maintenance of ion homeostasis in the cochlear fluids (Kikuchi et al., 2000). Among the various channels and junctions in these cochlear components, the gap junction is particularly important because it is involved in the maintenance of high potassium levels in endolymph; the mutation of genes associated with the gap junction is the most frequent cause of congenital SNHL (Cohen-Salmon et al., 2002; Schütz et al., 2011). A recent study demonstrated benefits of an *in utero* gene transfer for the treatment of congenital SNHL associated with the gap junction in a mouse model (Miwa et al., 2013). Thanks to the progress in genetic testing for deafness genes (Shearer et al., 2013), the number of patients with mutations in gap junction-associated genes will increase. In addition, the screening systems for deafness genes are becoming popular. Therefore, the restoration of gap junction-associated molecules will become a hot topic in the field of translational research of SNHL.

The first step in the conversion of sound stimuli into neural signals is a tilt of stereociliary bundles located at the top of hair cells. In the stereociliary bundles, there are several types of molecules that are essential for hearing, such as channels and junctions (Richardson et al., 2011; Fettiplace and Kim, 2014). In addition, the rootlet of the stereociliary bundles is also crucial for the maintenance of the stereocilia. Therefore, several types of molecules associated with the stereocilia can serve as therapeutic targets for SNHL. In the process of self-repair of hair cells, the restoration of the stereocilia is necessary for the functionality of hair cells. Therefore, studies aimed at the elucidation of the mechanisms behind the maintenance of the stereociliary bundle are required for increasing the efficacy of the self-repair approach.

Synaptic contacts between inner hair cells and spiral ganglion neurons are among the practical targets for the development of novel therapeutics for SNHL. Similar to the lateral wall and stereociliary bundle, the importance of synaptic contacts between inner hair cells and spiral ganglion neurons has been revealed by the discovery of the deafness genes associated with the ribbon synapse (Moser et al., 2013). The ribbon synapse is specific for the retina and inner ear and plays a crucial role in their functioning (Uthaiah and Hudspeth, 2010; Safieddine et al., 2012). Furthermore, the degeneration of synaptic contacts between inner hair cells and spiral ganglion neurons has been frequently implicated in the pathogenesis of SNHL caused by various factors, such as noise, aging, and ototoxic drugs (Kujawa and Liberman, 2009; Lin et al., 2011; Liu et al., 2013; Sergeyenko et al., 2013). Therefore, these synaptic contacts have become an important therapeutic target in SNHL.

Synaptic contacts between inner hair cells and spiral ganglion neurons have the capacity for spontaneous regeneration (Wang and Green, 2011). Therefore, the induction of their regeneration may be a practical treatment strategy. Previously, the search for effective protective agents for hair cells was the main strategy for the development of novel therapeutics for SNHL. In the coming decade, synapses between inner hair cells and spiral ganglion neurons are expected to become a center of attention in this type of translational research. In addition, the potential of gene therapy for restoration of VGLUT3, which is also an important molecule for the functionality of synaptic contacts between inner hair cells and spiral ganglion neurons, has been reported (Akil et al., 2009). Therefore, synaptic contacts between inner hair cells and spiral ganglion neurons will become a target of attention for the translation of gene therapy for the treatment of hearing.

## FUTURE PERSPECTIVES

In this study, we reviewed the present status and feasibility of the following four strategies for the induction of regeneration in mammalian cochlea: self-repair, transdifferentiation, dedifferentiation, and transplantation. Studies on these strategies have resulted in substantial progress toward clinical applications. In particular, novel self-repair techniques have already been tested for safety and efficacy in clinical trials.

For further acceleration of the exploration of novel therapeutics, the establishment of new screening systems may be the key challenge. Some studies demonstrated the efficacy of zebrafish models for this purpose (Esterberg et al., 2013). However, the drugs that have been identified and characterized using zebrafish screening systems have yet to be applied to clinical use.

Another promising screening tool is human iPS cells. The otic induction of pluripotent stem cells was recently reported (Oshima et al., 2010; Koehler et al., 2013; Ronaghi et al., 2014). Therefore, in theory, it is possible to generate some phenotypes of inner ear cells, especially sensory hair cells, from human iPS cells. Human iPS cell-derived cochlear cells could become a useful tool for the assessment of the efficacy of SNHL drugs and identification of differences in pharmacological effects among species. At present, the efficiency of otic induction by the published methods is inadequate, i.e., these techniques cannot generate sufficient numbers of inner ear cells for translational research. Further studies are required to refine the induction methods that can be utilized for research of disease-specific iPS cells.

The development of relevant diagnostic methods is also important. To fully utilize the detailed information generated by the studies of human deafness genes, clinical researchers require more focused and accurate diagnostic tools. At present, the functionality of outer hair cells is evaluated by otoacoustic emission. Recent studies have demonstrated that the amplitude of wave 1 in ABRs is closely related to the level of the degeneration of synaptic contacts between inner hair cells and spiral ganglion neurons (Kujawa and Liberman, 2009; Sergeyenko et al., 2013). The clinical utility of the measurement of ABR wave 1 amplitudes will be examined in the near future. In addition, with respect to functional analysis, more detailed morphological studies of cochleae are worthwhile. The benefits of an optical coherence tomograph have been recently evaluated (Gao et al., 2011, 2013; Kakigi et al., 2013; Tona et al., 2014b).

## Conflict of Interest Statement

The author declares that the research was conducted in the absence of any commercial or financial relationships that could be construed as a potential conflict of interest.
